# 2-Amino-4-methyl­benzene­sulfonamide

**DOI:** 10.1107/S1600536809037271

**Published:** 2009-09-26

**Authors:** Shao-Song Qian, Hao Hu, Hong-You Cui

**Affiliations:** a School of Life, ShanDong University of Technology, ZiBo 255049, People’s Republic of China; b School of Chemical Engineering, ShanDong University of Technology, ZiBo 255049, People’s Republic of China

## Abstract

In the crystal of the title compound, C_7_H_10_N_2_O_2_S, the mol­ecules are linked by two strong N—H⋯O hydrogen bonds. The mol­ecular structure is stabilized by an intra­molecular N—H⋯O hydrogen bond. The C/S/N plane makes a dihedral angle of 69.7 (2)° with the aromatic ring plane.

## Related literature

For the anti­convulsant activity of the title compound and its derivatives, see: Monzani *et al.* (1985 ▶); Tait *et al.* (1993 ▶). For hydrogen-bond motifs, see: Bernstein *et al.* (1995 ▶).
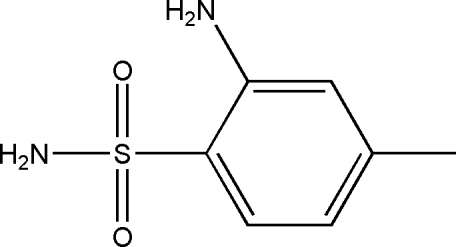


## Experimental

### 

#### Crystal data

C_7_H_10_N_2_O_2_S
*M* *_r_* = 186.23
Monoclinic, 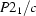

*a* = 9.873 (5) Å
*b* = 9.151 (4) Å
*c* = 10.408 (5) Å
β = 114.689 (6)°
*V* = 854.4 (7) Å^3^
*Z* = 4
Mo *K*α radiation
μ = 0.34 mm^−1^
*T* = 273 K
0.16 × 0.13 × 0.10 mm

#### Data collection

Bruker SMART CCD area-detector diffractometer
Absorption correction: multi-scan (*SADABS*; Sheldrick, 1996 ▶) *T*_min_ = 0.948, *T*_max_ = 0.967
4115 measured reflections
1449 independent reflections
1338 reflections with *I* > 2σ(*I*)
*R*_int_ = 0.017

#### Refinement

*R*[*F* ^2^ > 2σ(*F* ^2^)] = 0.047
*wR*(*F* ^2^) = 0.132
*S* = 1.10
1449 reflections
110 parameters
H-atom parameters constrained
Δρ_max_ = 0.56 e Å^−3^
Δρ_min_ = −0.41 e Å^−3^

### 

Data collection: *SMART* (Bruker, 2004 ▶); cell refinement: *SAINT* (Bruker, 2004 ▶); data reduction: *SAINT*; program(s) used to solve structure: *SHELXS97* (Sheldrick, 2008 ▶); program(s) used to refine structure: *SHELXL97* (Sheldrick, 2008 ▶); molecular graphics: *SHELXTL* (Sheldrick, 2008 ▶); software used to prepare material for publication: *SHELXTL*.

## Supplementary Material

Crystal structure: contains datablocks global, I. DOI: 10.1107/S1600536809037271/bx2239sup1.cif

Structure factors: contains datablocks I. DOI: 10.1107/S1600536809037271/bx2239Isup2.hkl

Additional supplementary materials:  crystallographic information; 3D view; checkCIF report

## Figures and Tables

**Table 1:** Hydrogen-bond geometry (Å, °)

*D*—H⋯*A*	*D*—H	H⋯*A*	*D*⋯*A*	*D*—H⋯*A*
N2—H2*B*⋯O2^i^	0.86	2.15	3.003 (4)	174
N2—H2*A*⋯O1^ii^	0.86	2.27	2.975 (4)	139
N1—H1*B*⋯O2	0.86	2.60	3.080 (5)	117

Symmetry codes: (i) 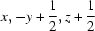; (ii) 
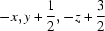
.

**Fig. 1.**
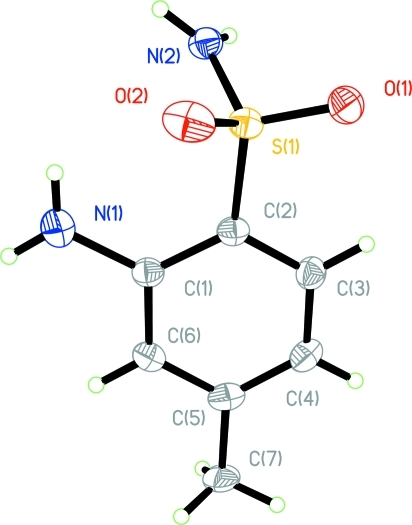
The structure of (I), showing the atom-labelling scheme. Displacement ellipsoids are drawn at the 35% probability level.

**Fig. 2.**
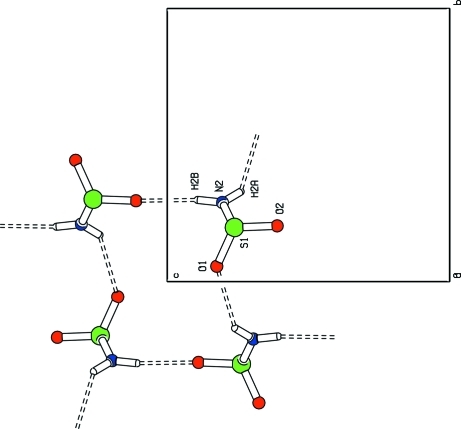
A portion of the packing diagram for (I) showing graph-set motif R44(16) ring. For the sake clarity the atoms not involved in the motif shown have been omitted Symmetry codes: (i) x, -y+1/2, z+1/2; (ii)-x,y+1/2,-z+3/2.

## supplementary crystallographic information

### Comment

The title compound (I), Figure1, was prepared and tested for anticonvulsant activity in mice, (Monzani, *et al.*,1985). In addition, its derivatives was studied using indomethacin as a reference drug (Tait, *et al.*,1993). We report here its crystal and molecular structure. The molecules are linked by two strong N—H···O generating a graph-set motif R^4^_4_(16) ring (Bernstein, *et al.*, 1995) and is stabilized by one N—H···O intramolecular hydrogen bonds (Table1, Figure 2). The C2/S1/N2 plane makes a dihedral angle of 69.7 (2)° with the aromatic ring plane.

### Experimental

The title compound was synthesized according to method described by Monzani, *et al.,1985.T*he single crystals suitable for X-ray diffraction were obtained by spontaneous evaporation of the solvent.

### Refinement

All the H atoms attached to C atoms were placed in geometrical positions and constrained to ride on their parent atoms with C—H distance in the range 0.93–0.98 Å, They were treated as riding atoms, with *U*_iso_(H) = 1.2*U*_eq_(C).

### Crystal data

**Table:**

C_7_H_10_N_2_O_2_S	*F*(000) = 392
*M**_r_* = 186.23	*D*_x_ = 1.448 Mg m^−^^3^
Monoclinic, *P*2_1_/*c*	Mo *K*α radiation, λ = 0.71073 Å
Hall symbol: -P 2ybc	Cell parameters from 3354 reflections
*a* = 9.873 (5) Å	θ = 2.2–28.2°
*b* = 9.151 (4) Å	µ = 0.34 mm^−^^1^
*c* = 10.408 (5) Å	*T* = 273 K
β = 114.689 (6)°	Block, colorless
*V* = 854.4 (7) Å^3^	0.16 × 0.13 × 0.10 mm
*Z* = 4	

### Data collection

**Table:**

Bruker SMART CCD area-detector diffractometer	1449 independent reflections
Radiation source: fine-focus sealed tube	1338 reflections with *I* > 2σ(*I*)
graphite	*R*_int_ = 0.017
φ and ω scans	θ_max_ = 25.1°, θ_min_ = 3.1°
Absorption correction: multi-scan (*SADABS*; Sheldrick, 1996)	*h* = −11→11
*T*_min_ = 0.948, *T*_max_ = 0.967	*k* = −10→10
4115 measured reflections	*l* = −12→9

### Refinement

**Table:**

Refinement on *F*^2^	Secondary atom site location: difference Fourier map
Least-squares matrix: full	Hydrogen site location: inferred from neighbouring sites
*R*[*F*^2^ > 2σ(*F*^2^)] = 0.047	H-atom parameters constrained
*wR*(*F*^2^) = 0.132	*w* = 1/[σ^2^(*F*_o_^2^) + (0.0721*P*)^2^ + 0.701*P*] where *P* = (*F*_o_^2^ + 2*F*_c_^2^)/3
*S* = 1.10	(Δ/σ)_max_ < 0.001
1449 reflections	Δρ_max_ = 0.56 e Å^−^^3^
110 parameters	Δρ_min_ = −0.41 e Å^−^^3^
0 restraints	Extinction correction: *SHELXL97* (Sheldrick, 2008), Fc^*^=kFc[1+0.001xFc^2^λ^3^/sin(2θ)]^-1/4^
Primary atom site location: structure-invariant direct methods	Extinction coefficient: 0.047 (9)

### Special details

**Table:**

Geometry. All e.s.d.'s (except the e.s.d. in the dihedral angle between two l.s. planes) are estimated using the full covariance matrix. The cell e.s.d.'s are taken into account individually in the estimation of e.s.d.'s in distances, angles and torsion angles; correlations between e.s.d.'s in cell parameters are only used when they are defined by crystal symmetry. An approximate (isotropic) treatment of cell e.s.d.'s is used for estimating e.s.d.'s involving l.s. planes.
Refinement. Refinement of *F*^2^ against ALL reflections. The weighted *R*-factor *wR* and goodness of fit *S* are based on *F*^2^, conventional *R*-factors *R* are based on *F*, with *F* set to zero for negative *F*^2^. The threshold expression of *F*^2^ > σ(*F*^2^) is used only for calculating *R*-factors(gt) *etc*. and is not relevant to the choice of reflections for refinement. *R*-factors based on *F*^2^ are statistically about twice as large as those based on *F*, and *R*- factors based on ALL data will be even larger.

### Fractional atomic coordinates and isotropic or equivalent isotropic displacement parameters (Å^2^)

**Table:**

	*x*	*y*	*z*	*U*_iso_*/*U*_eq_	
S1	0.13944 (9)	0.19821 (9)	0.76214 (8)	0.0361 (4)	
O1	0.1511 (3)	0.0560 (3)	0.8246 (3)	0.0533 (8)	
O2	0.0800 (3)	0.2040 (4)	0.6109 (3)	0.0603 (9)	
N1	0.2315 (4)	0.5060 (4)	0.6773 (4)	0.0604 (10)	
H1A	0.2566	0.5867	0.6510	0.073*	
H1B	0.1391	0.4815	0.6450	0.073*	
N2	0.0260 (3)	0.2896 (3)	0.8048 (3)	0.0412 (7)	
H2A	−0.0514	0.3293	0.7402	0.049*	
H2B	0.0426	0.2990	0.8924	0.049*	
C1	0.3488 (4)	0.4078 (4)	0.7793 (3)	0.0357 (7)	
C2	0.3186 (3)	0.2754 (4)	0.8318 (3)	0.0342 (7)	
C3	0.4324 (4)	0.1967 (4)	0.9361 (4)	0.0396 (8)	
H3	0.4098	0.1121	0.9727	0.048*	
C4	0.5787 (4)	0.2433 (4)	0.9859 (4)	0.0443 (9)	
H4	0.6543	0.1920	1.0571	0.053*	
C5	0.6107 (3)	0.3669 (4)	0.9282 (4)	0.0417 (8)	
C6	0.4979 (4)	0.4501 (4)	0.8300 (4)	0.0406 (8)	
H6	0.5221	0.5362	0.7969	0.049*	
C7	0.7610 (3)	0.4151 (5)	0.9737 (4)	0.0497 (9)	
H7A	0.7795	0.4947	1.0389	0.074*	
H7B	0.7771	0.4471	0.8933	0.074*	
H7C	0.8276	0.3360	1.0193	0.074*	

### Atomic displacement parameters (Å^2^)

**Table:**

	*U*^11^	*U*^22^	*U*^33^	*U*^12^	*U*^13^	*U*^23^
S1	0.0355 (6)	0.0364 (6)	0.0356 (6)	−0.0058 (3)	0.0140 (4)	−0.0071 (3)
O1	0.0470 (15)	0.0348 (14)	0.0722 (18)	−0.0059 (11)	0.0191 (13)	−0.0030 (12)
O2	0.0540 (16)	0.086 (2)	0.0376 (14)	−0.0211 (14)	0.0163 (12)	−0.0185 (13)
N1	0.056 (2)	0.063 (2)	0.062 (2)	0.0026 (16)	0.0245 (17)	0.0197 (17)
N2	0.0373 (16)	0.0455 (17)	0.0419 (15)	0.0014 (12)	0.0174 (13)	0.0004 (12)
C1	0.0368 (16)	0.0390 (17)	0.0348 (15)	0.0007 (13)	0.0184 (13)	−0.0019 (13)
C2	0.0329 (16)	0.0357 (16)	0.0352 (16)	−0.0012 (12)	0.0155 (13)	−0.0053 (12)
C3	0.0414 (18)	0.0327 (17)	0.0435 (18)	0.0024 (13)	0.0165 (15)	0.0001 (13)
C4	0.0360 (18)	0.0431 (19)	0.0474 (19)	0.0067 (14)	0.0109 (15)	−0.0027 (16)
C5	0.0313 (17)	0.047 (2)	0.0467 (18)	−0.0029 (14)	0.0165 (14)	−0.0113 (15)
C6	0.0395 (17)	0.0420 (18)	0.0443 (18)	−0.0051 (14)	0.0213 (15)	−0.0005 (15)
C7	0.0246 (16)	0.058 (2)	0.062 (2)	−0.0056 (15)	0.0139 (16)	−0.0010 (18)

### Geometric parameters (Å, °)

**Table:**

S1—O2	1.433 (3)	C2—C3	1.393 (5)
S1—O1	1.438 (3)	C3—C4	1.383 (5)
S1—N2	1.602 (3)	C3—H3	0.9300
S1—C2	1.756 (3)	C4—C5	1.378 (6)
N1—C1	1.500 (5)	C4—H4	0.9300
N1—H1A	0.8600	C5—C6	1.383 (5)
N1—H1B	0.8600	C5—C7	1.426 (4)
N2—H2A	0.8600	C6—H6	0.9300
N2—H2B	0.8600	C7—H7A	0.9600
C1—C6	1.395 (5)	C7—H7B	0.9600
C1—C2	1.412 (5)	C7—H7C	0.9600
			
O2—S1—O1	116.68 (18)	C4—C3—C2	120.6 (3)
O2—S1—N2	105.80 (18)	C4—C3—H3	119.7
O1—S1—N2	106.29 (16)	C2—C3—H3	119.7
O2—S1—C2	108.58 (16)	C5—C4—C3	118.9 (3)
O1—S1—C2	107.49 (16)	C5—C4—H4	120.5
N2—S1—C2	112.08 (16)	C3—C4—H4	120.5
C1—N1—H1A	120.0	C4—C5—C6	120.9 (3)
C1—N1—H1B	120.0	C4—C5—C7	120.3 (3)
H1A—N1—H1B	120.0	C6—C5—C7	118.7 (4)
S1—N2—H2A	120.0	C5—C6—C1	121.5 (3)
S1—N2—H2B	120.0	C5—C6—H6	119.2
H2A—N2—H2B	120.0	C1—C6—H6	119.2
C6—C1—C2	116.9 (3)	C5—C7—H7A	109.5
C6—C1—N1	118.9 (3)	C5—C7—H7B	109.5
C2—C1—N1	124.3 (3)	H7A—C7—H7B	109.5
C3—C2—C1	120.9 (3)	C5—C7—H7C	109.5
C3—C2—S1	117.4 (3)	H7A—C7—H7C	109.5
C1—C2—S1	121.6 (3)	H7B—C7—H7C	109.5

### Hydrogen-bond geometry (Å, °)

**Table:**

*D*—H···*A*	*D*—H	H···*A*	*D*···*A*	*D*—H···*A*
N2—H2B···O2^i^	0.86	2.15	3.003 (4)	174
N2—H2A···O1^ii^	0.86	2.27	2.975 (4)	139
N1—H1B···O2	0.86	2.60	3.080 (5)	117

Symmetry codes: (i) *x*, −*y*+1/2, *z*+1/2; (ii) −*x*, *y*+1/2, −*z*+3/2.
